# Discriminating legitimate oscillations from broadband transients

**DOI:** 10.1186/1471-2202-14-S1-P286

**Published:** 2013-07-08

**Authors:** Vasile V Moca, Raul C Mureşan

**Affiliations:** 1 Department of Experimental and Theoretical Neuroscience, Center for Cognitive and Neural Studies (Coneural), Romanian Institute of Science and Technology, Cluj-Napoca, Cluj, 400487, Romania

## 

Neural oscillations are one of the most prominent characteristics of brain activity. Unfortunately, quantification of oscillations is not always straightforward. For spiking activity the pulsed nature of the binary signal makes spectral methods difficult to apply [1]. Counterintuitively, difficulties arise also for the case of continuous data (such as the local field potentials - LFPs, or the electroencephalogram - EEG) because their estimated spectra can be contaminated by broadband transient (BT) noise. Notably, muscle and ocular artifacts are known to produce BTs [2] that overlap with the gamma band (30-80 Hz), which is particularly relevant for information processing and seems to be correlated to conscious states. To address the issue of BTs in EEG, independent component analysis (ICA) has been employed for artifact treatment. However ICA is suitable only for multichannel recordings and may not be always satisfactory [3]. Eye-tracking data has been shown to help ICA cope better with eye artifacts [4].

Here we intend to explore an alternative approach to separating legitimate oscillations from BTs. This approach is based on previous work on oscillations in binary spiking data, where we have proposed a method relying on the spectrum of the autocorrelation function (ACF), namely the oscillation score (OS) [1]. OS takes into account particularities of ACF in order to isolate the truly periodic components which are then quantified by spectral analysis of the modified ACF. Most notably, the OS removes the central peak of the ACF, whose presence is not a characteristic of oscillatory activity. The central peak is very large, usually narrow and contaminates the ACF spectrum with broadband noise. After removing the central peak, the OS is capable to correctly quantify the strength of oscillations eliminating broadband contamination and overcoming difficulties associated with other methods [1].

For continuous signals like EEG or LFP, one should note that non-periodic spike-like transients (e.g., microsaccadic artifacts) should influence only the central peak of the ACF. Following the same idea as in OS, the influence of BTs could be mitigated by careful manipulation of the central peak. One legitimate question is whether the ACF central peak should be removed as in the case of OS, rescaled, or left intact. Here, we show that an extremely large central peak indicates BTs and that its width reflects the width of the transients. By considering the envelope of the ACF computed on the side-lobes, we show how to correctly handle the central peak (rescaling) to remove the influence of non-periodic transients with minimal distortion to the ACF's spectrum.

According to the Wiener-Khinchin theorem, the Fourier transform of the ACF is the power spectrum of the signal. Therefore, we show that by applying an appropriate correction to the ACF central peak one is able to estimate the power spectrum of the signal while minimizing the influence of BTs. The method presented here and the OS demonstrate that mixed correlation-spectral approaches can offer a unified framework for robust quantification of oscillations in both continuous (LFP, EEG) and discrete spiking data.
